# The CSF Vancomycin Concentration in Patients With Post-operative Intracranial Infection Can Be Predicted by the WBCs to Total Cells Ratio and the Serum Trough Concentration

**DOI:** 10.3389/fneur.2022.893089

**Published:** 2022-05-13

**Authors:** Ming-Chao Fan, Jia-Lin Sun, Jian Sun, Jun-Wei Ma, Nian Wang, Wei Fang

**Affiliations:** ^1^Department of Neurosurgery, the Affiliated Hospital of Qingdao University, Qingdao, China; ^2^Department of Neurosurgical Intensive Care Unit, the Affiliated Hospital of Qingdao University, Qingdao, China; ^3^Department of Pharmacy, the Affiliated Hospital of Qingdao University, Qingdao, China; ^4^Department of Critical Care Medicine, Shandong Provincial Hospital Affiliated to Shandong First Medical University, Jinan, China

**Keywords:** vancomycin, intracranial infection, blood-brain barrier, cerebrospinal fluid, serum trough concentration

## Abstract

**Background:**

The pharmacokinetics of vancomycin in cerebrospinal fluid (CSF) is an important basis for evaluating the bactericidal effect. The accuracy of using serum vancomycin concentrations only to estimate the CSF concentrations remains controversial, may lead to underdosing.

**Objectives:**

The aims of this study were to evaluate the vancomycin exposure in CSF, investigate the factors affecting the vancomycin blood–brain barrier (BBB) penetration, and to establish the prediction model of vancomycin concentration in CSF.

**Methods:**

Eligible patients were included and given a standard dose of vancomycin. At the fifth dose, the blood and CSF samples were collected 0.5 h before the start of infusion of vancomycin, and 1, 2, 3, and 8 h from the start of infusion, and were measured by the enzyme-multiplied immunoassay technique using the Siemens Viva-E Drug Testing System.

**Results:**

The AUC_CSF/*serum*_ of patients with intracranial infection was higher than that of patients without (*p* = 0.001). The CSF concentration was relatively stable between dosing periods (*p* = 0.095). The area under the concentration–time curve (AUC) ratio of CSF to serum (AUC_CSF/serum_) in patients with intracranial infection ranged from 15.1 to 80.1% (33.23 ± 19.31%; median, 26.25%). The CSF vancomycin AUC levels were affected by the serum trough concentration (*B*: 5.23 ± 2.36, *t* = 2.22, *p* = 0.039), and were mainly affected by the CSF white blood cells (WBCs)/total cells (*B*: 113.96 ± 35.10, *t* = 3.25, *p* = 0.004) (Y = −17.86 + 5.23 × serum trough concentration + 113.96 × CSF [WBCs/total cells]; *R*^2^ = 0.473, *F* = 8.542, *p* = 0.002).

**Conclusions:**

After intravenous administration of vancomycin, the CSF concentration curve was fluctuated gently. The CSF vancomycin concentration in patients with postoperative intracranial infection can be predicted by the WBCs to total cells ratio and the serum trough concentration, and help to adjust the administration of vancomycin.

## Introduction

Postoperative intracranial bacterial infection is a well-known potential life-threatening complication in neurosurgical therapy, and it often seriously affects the clinical prognosis, and increases the mortality (1–3). Although the most important treatment for bacterial infections is adequate source control, antibiotic treatment is also one of the most important measures.

Vancomycin is a hydrophilic, macromolecular glycopeptide antibiotic, and its main elimination route is renal excretion (4). Its bactericidal activity is mainly against Gram-positive (G^+^) pathogens (5, 6). Most of the bacteria-infected cases after craniocerebral operation are G^+^ pathogens (7). Vancomycin serves as a first-line empiric antibiotic for intracranial infection after surgery (8).

There is insufficient evidence to recommend the widespread use of local intraventricular antibiotic therapy for intracranial infections. It should only be considered if the patients respond poorly to systemic antimicrobial therapy, and in patients with multi-organ dysfunction in intensive care (7, 9).

The effectiveness of antimicrobial therapy depends on the concentration level of the drug in the target organ (10). For patients with postoperative intracranial infection, the exposure of cerebrospinal fluid (CSF) vancomycin determines its bactericidal effect (11). Intravenous injection (I.V.) vancomycin must enter the brain tissue through the blood–brain barrier (BBB). Because of its hydrophilicity and macromolecules, its penetration into the CSF is subject to many restrictions. There are multiple uncertainties which may influence the penetration of vancomycin into CSF, not only the serum vancomycin concentration but also the extent of BBB disruption (11). In the treatment of intracranial bacterial infections, vancomycin use is based on the hypothesis that the BBB is disrupted when the central nervous system is inflamed (12). Although it has been known that the integrity of the BBB is disruption during intracranial infection, and vancomycin penetration is increased. We still lack the quantitative relationship between vancomycin CSF and serum concentration, and the characteristics of CSF vancomycin concentration curve. The accuracy of using serum vancomycin concentrations only to estimate CSF concentrations remains controversial, may lead to underdosing (13, 14). The pharmacokinetics (PK) of vancomycin in CSF is an important basis for evaluating the bactericidal effect, but rarely found in previous literatures, and large discrepancy in results.

It is of great significance to find potential indicators in routine detection indicators to predict drug concentration and exposure in CSF. The aims of this study were to evaluate vancomycin exposure in CSF, investigate the factors affecting the vancomycin BBB penetration, and to establish the prediction model of vancomycin concentration in CSF.

## Methods

### Patient Population

This prospective observational study was performed in the Affiliated Hospital of Qingdao University from March 2018 to December 2019. All consecutive patients were hospitalized in the Neurosurgical Intensive Care Unit with proven or suspected postoperative intracranial infection were enrolled.

Postoperative intracranial infection was defined as at least two of the following criteria: (A) clinical signs and symptoms: fever (>38°C) and meningeal irritation sign (+); (B) a positive result of CSF culture; (C) two or more changes in CSF specimens: (1) CSF glucose <2.2 mmol/L or <40% of serum glucose; (2) protein concentrations >600 mg/L; (3) white blood cells (WBCs) count more than 1,000 × 10^6^/L or WBCs/total cells >1%, with coenocyte predominance >50%.

Exclusion criteria included: (1) the patient or guardian refused to participate; (2) the patient with hypersensitivity to vancomycin; (3) the patient received vancomycin treatment by any means within 1 month; (4) the patient with abnormal hepatic and renal function; (5) CSF could not be collected; (6) age < 18 years old; (7) the result of CSF culture was G^−^ bacilli.

Basic demographic data of the patients were obtained from their guardians. The body temperature, weight, and height of the patients were collected before vancomycin treatment. Collected and recorded the laboratory parameters on the same day, such as blood WBCs, serum albumin, creatinine clearance rate (CLcr), serum procalcitonin (PCT), serum protein, and serum glucose.

The estimated creatinine clearance was calculated based on the Cockcroft–Gault formula. Infusion reaction and anaphylaxis were monitored during each infusion.

### Drug Administration and Samples Collection

All patients received I.V. vancomycin (Vancocin, Vianex S.A.) alone or in combination with meropenem. Vancomycin was administrated I.V. 1,000 mg every 12 h (0.9% NaCl 250 ml dilution, continuous infused 2 h with a syringe pump) as the recommended daily dose for adult patients with normal renal function. No patients received corticosteroids during anti-infection treatment. Samples of blood and CSF were collected at the fifth dose (day 3). At least 2 ml of venous blood and 2 ml of CSF were collected 0.5 h before the start of infusion of vancomycin, and 1, 2, 3, and 8 h from the start of infusion. CSF samples were obtained by a temporary lumbar catheterization. About 2 ml of CSF remaining in the drainage tube was discarded, and 2 ml of fresh CSF was collected. The blood and CSF samples were collected using normal biochemical test tubes.

At the fifth CSF collection, appropriate amount of CSF was collected for CSF biochemical analysis, cytological analysis, PCT analysis, and microbial culture. Bacteriological antibiotic sensitivity test was determined using the Vitek-2 Compact automatic system (Biomerieux Inc., France). The temporary lumbar tube was removed or retained according to treatment needs after the collection of CSF samples.

### Drug Concentration Detection

All blood and CSF samples were centrifuged and the supernatants were collected for assay. The vancomycin concentration in serum and CSF was measured by the enzyme-multiplied immunoassay technique using the Siemens Viva-E Drug Testing System with the Emit 2,000 Vancomycin Assay Test Kit (Siemens Healthcare Diagnostic Inc. Germany). This method had a calibration range between 2.0 and 50.0 μg/ml, with a lowest limit of 2.0 μg/ml. The variation coefficients were <10% for both Intra- and inter-assay. All samples were tested by the same pharmaceutical technician and reviewed by another.

### Pharmacokinetic Analysis

The PK parameters including drug clearance (CL_z_) and area under the 24-h concentration–time curve (AUC) were calculated by a non-compartmental analysis method using the DAS 3.0.2 pharmacokinetic program. As observable parameters, the time to reach the maximum concentration (*T*_*max*_) and the maximum concentration (*C*_*max*_) were obtained directly. Vancomycin concentration–time curve was measured and the relationship between vancomycin CSF and serum level was calculated.

### Statistical Analysis

Statistical analysis was performed using SPSS 24.0 (SPSS Inc., Chicago, Illinois, USA). All quantitative data were expressed as mean ± standard deviation. The student's *t*-tests for small samples (N ≤ 22, assuming a normal distribution), ANOVA for repeated measurement data was used to assess differences between variables. Pearson's correlation analysis was used to assess the correlation between two variables. Multiple linear regression was used to analyze the relationship between multiple factors. The *p*-values of < 0.05 were considered as statistically significant.

The covariance analysis of vancomycin concentration in CSF and serum and AUC of patients in the two groups was conducted with body weight as covariable, and it was found that the significant factors of body weight were all >0.05, so covariance analysis was not applicable.

The maximum limit of CSF protein was 3,000 mg/L, the lowest limit of CSF glucose was 1.1 mmol/L, and the lowest limit of CSF vancomycin concentration was 2.0 μg/ml in the laboratory department of our hospital. Therefore, for statistical purposes, CSF protein >3,000 mg/L was considered to be 3,000 mg/L, CSF glucose ≤1.1 mmol/L was regarded as 1.1 mmol/L, and CSF vancomycin concentration <2.0 μg/ml was considered to be 0 μg/ml for statistical purposes.

## Results

A total of 22 patients (13 men and 9 women; age: range 18–83 years, mean 57.95 ± 14.96 years) with 110 blood samples and 110 CSF samples were included in this study (Table 1). Twelve of these patients were clinically diagnosed as postoperative intracranial infection (6 men and 6 women; age: range 18–78 years, mean 55.83 ± 17.61 years), and 10 patients had high risk factors of infection but ruled out eventually (6 men and 4 women; age: range 45–83 years, mean 60.50 ± 11.42 years). There were no significant differences, were found in age, sex, etiology, CLcr, serum albumin, body weight, height, or operation between the patients with and without intracranial infection (*p* > 0.05). All patients received standard pharmacological treatment for the primary disease, and there was no differences between-group. During the treatment period, no adverse reactions such as vancomycin infusion reaction, decreased WBCs, or renal impairment were observed. The clinical and bacteriological cures were achieved for all patients. No correlation was found between body weight and drug concentration or AUC (*p* > 0.05).

**Table 1:** The demographics of these 22 patients.

**Patients**	**Infection**	**Gender**	**Age (year)**	**Weight (Kg)**	**Serum trough concentration (μg/mL)**	**CSF trough concentration (μg/mL)**	**Serum AUC**	**CSF AUC**	**CSF WBCs (× 10^**9**^/L)**	**CSF PCT (ng/mL)**	**CSF proportion of polynuclear cells**	**CSF WBCs/total cells**
1	Yes	Male	51	90	12.50	7.00	372.23	196.00	26851.00	0.195	97.00	0.6910
2	Yes	Female	66	57	5.70	3.40	374.23	62.74	6597.00	0.164	84.50	0.6230
3	Yes	Female	50	60	7.80	3.60	322.80	65.17	17590.00	0.265	93.00	0.3280
4	Yes	Male	54	80	13.70	11.10	338.00	270.67	1612.00	0.140	80.00	0.5890
5	Yes	Female	78	72	19.40	6.40	368.26	106.98	1189.00	0.108	94.00	0.3050
6	Yes	Female	77	60	5.60	2.70	233.25	50.28	1927.00	0.098	92.00	0.2100
7	Yes	Male	35	102	10.50	4.50	426.01	72.90	1894.00	0.152	92.00	0.9980
8	Yes	Male	64	60	6.40	3.50	236.67	57.98	266.00	0.102	57.00	0.4000
9	Yes	Female	73	53	13.90	3.40	368.59	55.50	379.00	0.114	64.00	0.3970
10	Yes	Female	57	65	10.40	3.70	332.53	74.24	14118.00	0.057	92.60	0.6380
11	Yes	Male	18	62	4.80	2.10	222.55	62.33	4818.00	0.069	87.00	0.6160
12	Yes	Male	47	100	7.00	3.10	237.88	83.89	1508.00	0.132	94.00	0.4290
13	No	Male	66	70	5.70	2.20	192.08	33.94	654.00	0.060	72.00	0.0080
14	No	Male	58	75	4.60	2.10	224.49	37.90	153.00	0.151	98.00	0.0040
15	No	Male	83	72	10.90	3.00	335.22	61.19	1250.00	0.101	79.00	0.0090
16	No	Female	54	80	15.50	2.00	423.76	32.70	0.00	0.048	0.00	0.0000
17	No	Male	73	85	15.70	2.60	391.26	59.15	4.00	0.023	0.00	0.0000
18	No	Female	64	89	8.40	2.00	263.62	33.18	847.00	0.031	85.10	0.2200
19	No	Female	51	50	4.80	<2.00	282.90	0.00	7.00	0.024	0.00	0.0000
20	No	Male	45	72	4.70	<2.00	206.23	0.00	2.00	0.065	0.00	0.0000
21	No	Male	60	85	4.10	<2.00	205.21	0.00	79.00	0.042	0.00	0.0090
22	No	Female	51	50	12.80	<2.00	361.09	0.00	225.00	0.084	49.00	0.0060

**Figure 1:**
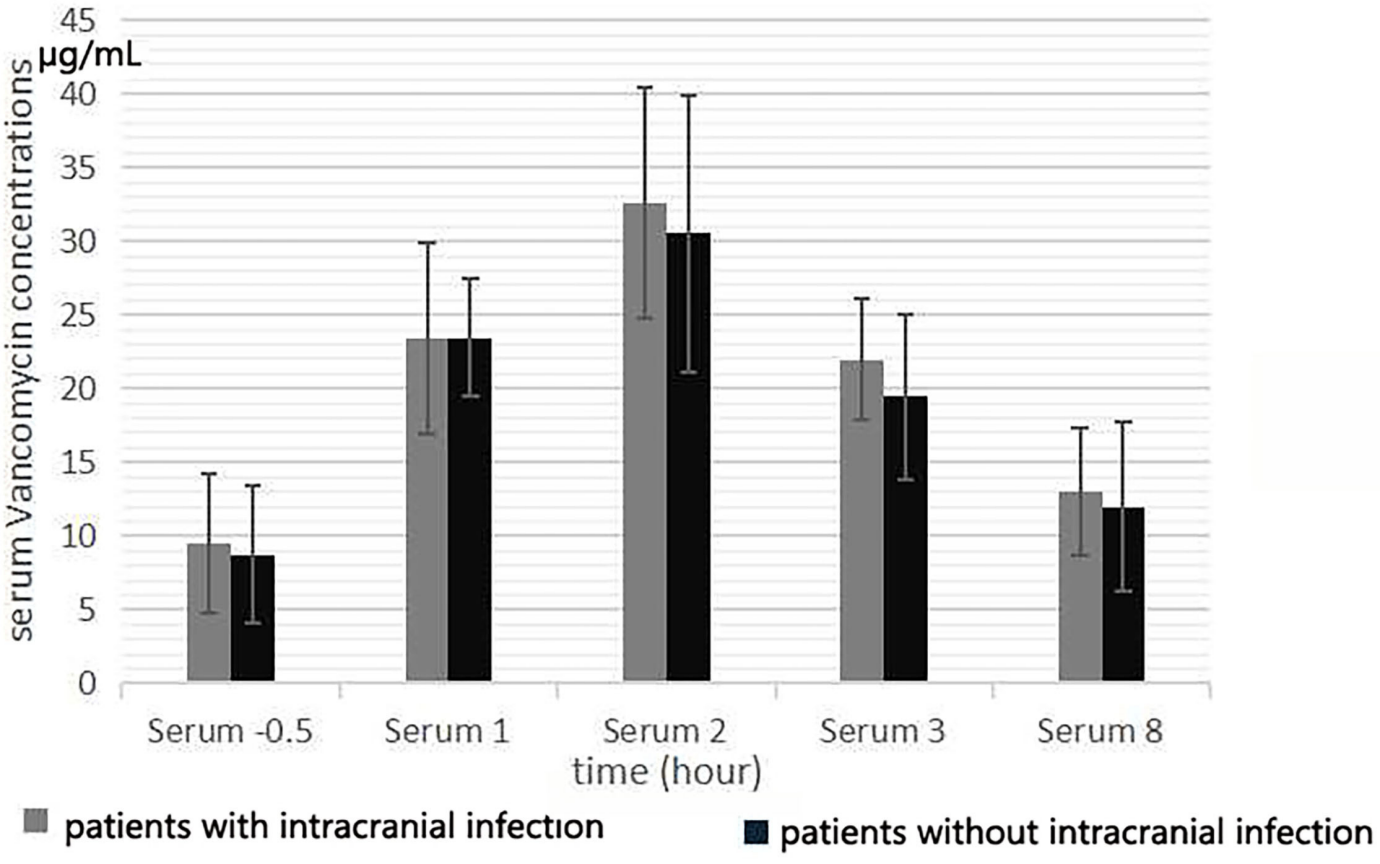
Serum vancomycin concentration at 0.5 h before the start of the fifth infusion, and 1, 2, 3, and 8 h from the start of infusion.

**Figure 2:**
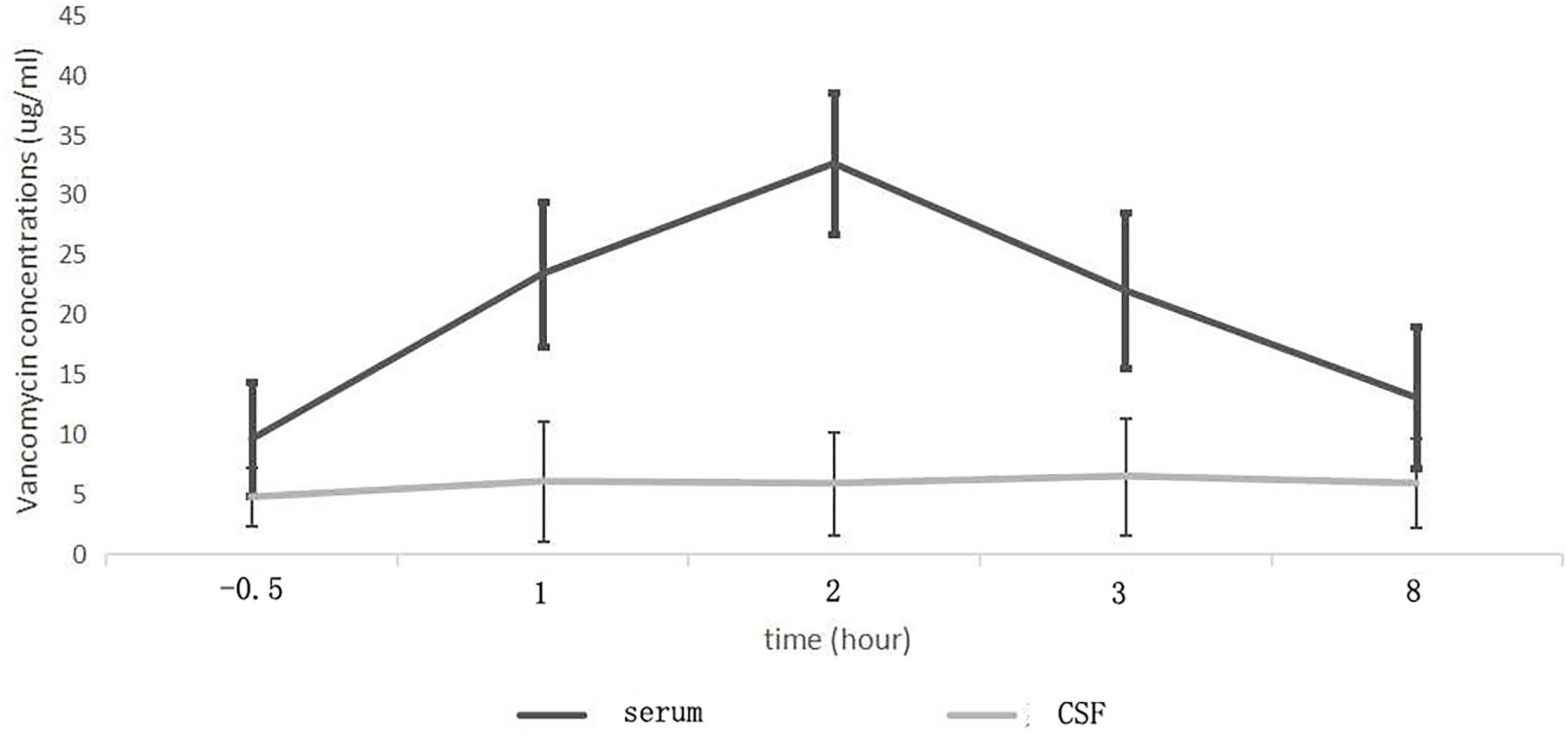
Mean serum and cerebrospinal fluid (CSF) vancomycin concentration-time curves in patients with intracranial infection.

The mean serum vancomycin concentration of patients evaluated during the fifth 12 h of treatment is presented in Figure 1. After intravenous infusion, the fluctuation of serum vancomycin concentration was remarkable, with obvious the peak and trough concentrations (Figure 2). There were significant differences in serum concentration at different collection times (*F* = 109.61, *p* < 0.001). The concentration at 0.5 h before the fifth dose was the serum vancomycin trough concentration. The *T*_*max*_ of the serum concentration was 2 h after the start of infusion of vancomycin. No significant differences were found in the serum vancomycin concentration between the patients with and without intracranial infection (*F* = 0.409, *p* = 0.530). The serum trough concentration levels were affected by the CLcr (B: −0.30 ± 0.009, *r* = −0.609, *R*^2^ = 0.371, *F* = 11.809, *p* = 0.003). The serum AUC was negatively correlated with CLcr (*r* = −0.469, *p* = 0.028).

Of the 12 patients with intracranial infection, there were 6 positive cultures of G^+^ bacteria (Table 2). The *MIC* value of only one strain of *Staphylococcus epidermidis* was 1 μg/ml, and the values of other five strains were all ≤ 0.5 μg/ml. The *MIC* ≤0.5 μg/ml was considered to be 0.5 μg/ml for statistical purposes. The CSF AUC_(0−24)_/*MIC* ratios ranged from 72.90 to 196.00 (median, 125.07) for these 6 patients.

**Table 2:** Distribution and characteristics of G^+^ bacteria in cerebrospinal fluid culture.

	**G+ bacteria**	**MIC μg/mL**	**AUC_**CSF**_**	**AUC_**CSF/MIC**_**
1	Staphylococcus hominis	≤0.5	62.74	125.48
2	Staphylococcus hominis	≤0.5	62.33	124.66
3	Staphylococcus epidermidis	1	72.90	72.90
4	Staphylococcus epidermidis	≤0.5	83.89	167.78
5	Enterococcus faecium	≤0.5	57.98	115.96
6	Staphylococcus aureus	≤0.5	98.00	196.00

**Figure 3:**
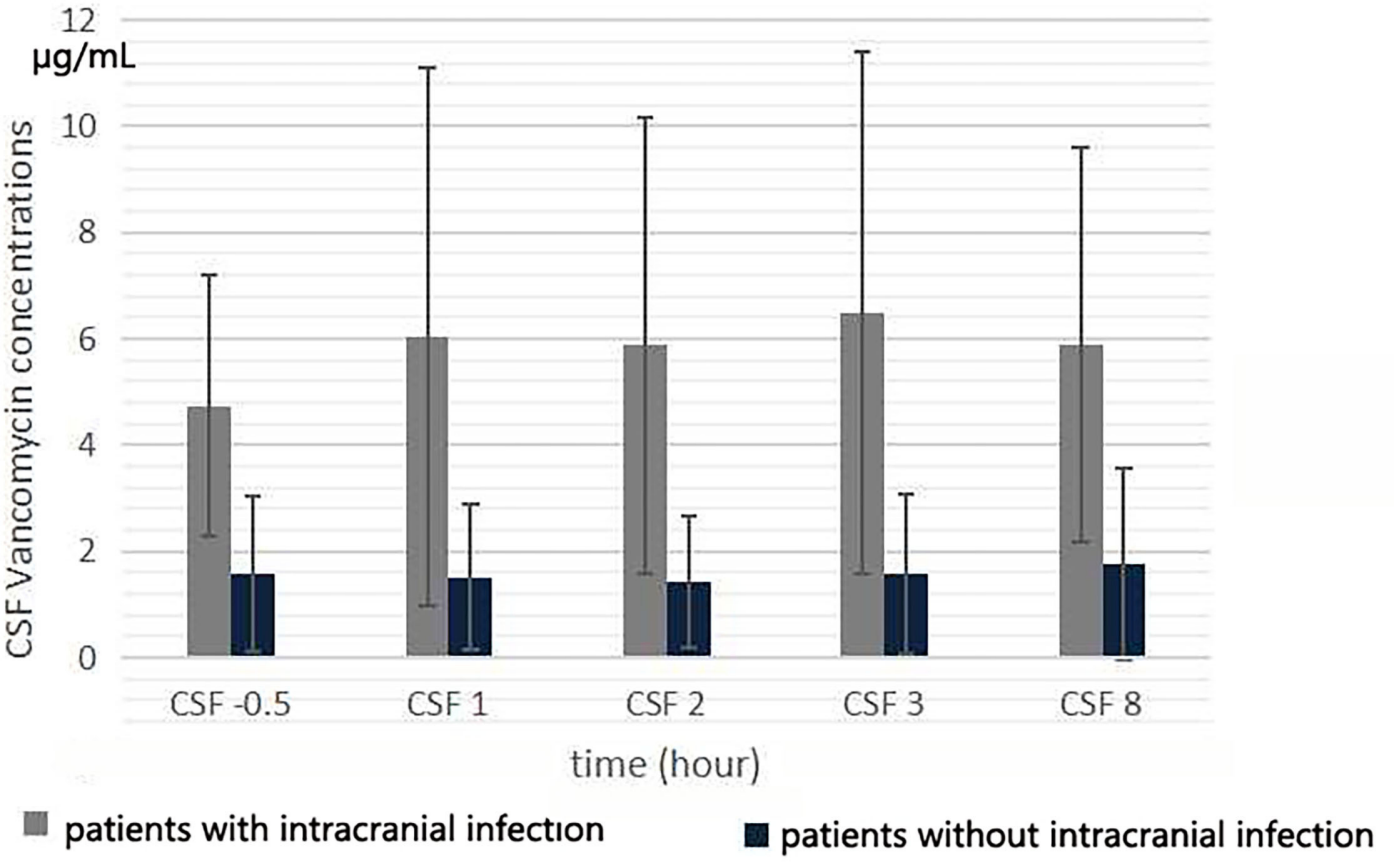
Cerebrospinal fluid vancomycin concentration at 0.5 h before the start of the fifth infusion, and 1, 2, 3, and 8 h from the start of infusion.

The CSF vancomycin concentration was <2.0 μg/ml in 4 patients without intracranial infection. The mean CSF vancomycin concentration of patients evaluated during the fifth 12 h of treatment is presented in Figure 3. After intravenous infusion, the fluctuation of CSF vancomycin concentration was small, and the peak and trough concentrations were not obvious (Figure 2). There was no significant differences in CSF concentration at different collection times (*F* = 2.051, *p* = 0.095). The CSF *C*_*max*_ of patients with intracranial infection was ranged from 3.6 to 20.5 μg/ml and of patients without intracranial infection ranged from 0 to 4.8 μg/ml (6.96 ± 4.98 μg/ml *vs*. 1.90 ± 1.83 μg/ml, *t* = 3.03, *p* = 0.007). The CSF trough levels of patients with and without intracranial infection were, respectively, ranged from 2.1 to 11.1 μg/ml and from 0 to 3.0 μg/ml (4.54 ± 2.51 μg/ml *vs*. 1.39 ± 1.23 μg/ml, *t* = 3.62, *p* = 0.002). Differences in CSF vancomycin concentration between patients with and without intracranial infection were significant (*F* = 9.857, *p* = 0.005).

**Figure 4:**
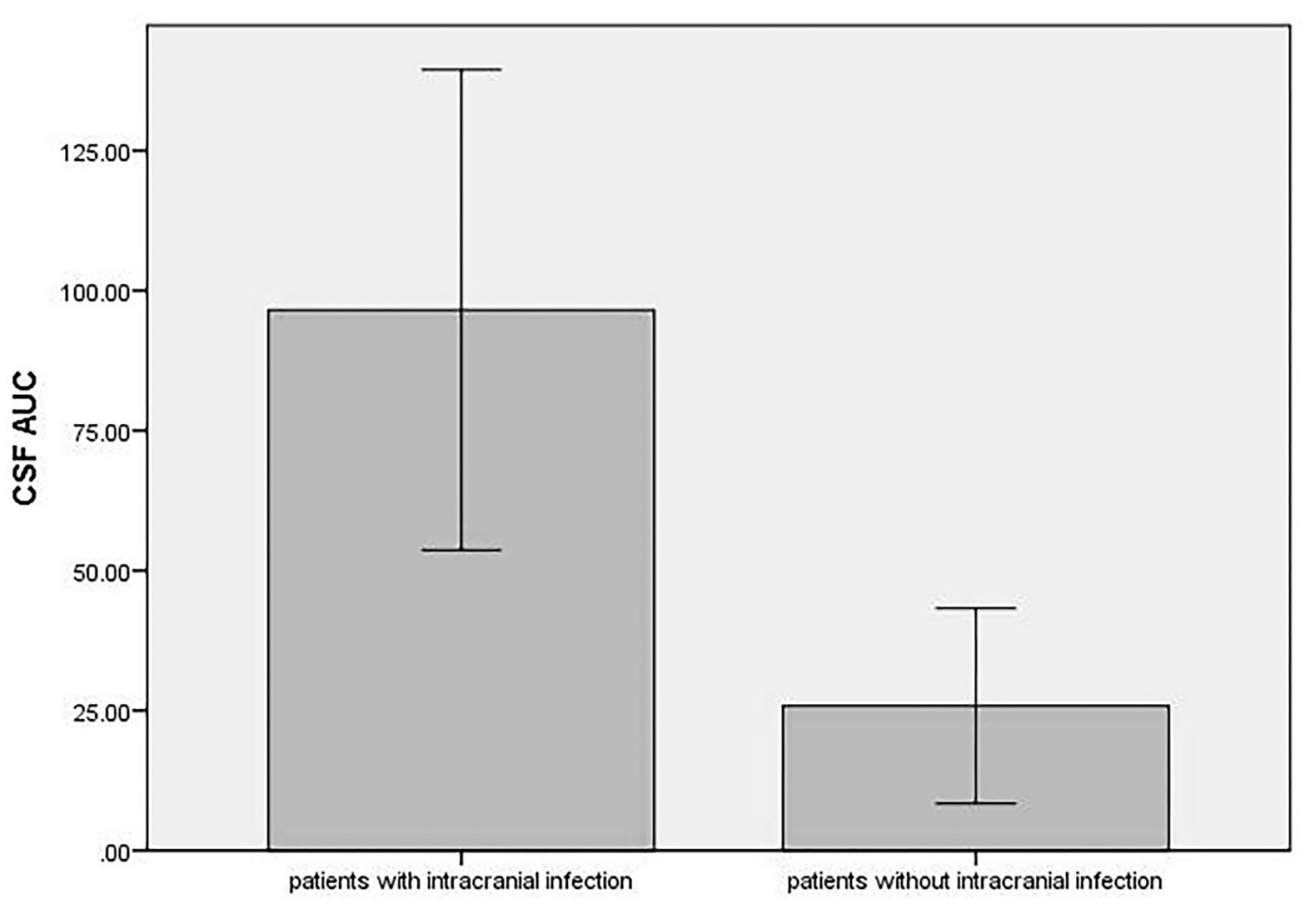
The CSF AUC in patients with and without intracranial infection.

The serum AUC of patients with and without intracranial infection, respectively, ranged from 222.55 to 426.01 μg/ml·h and from 192.08 to 423.76 μg/ml·h (319.42 ± 69.28 μg/ml·h *vs*. 288.59 ± 84.36 μg/ml·h, *t* = 0.94, *p* = 0.357). The CSF AUC of patients with intracranial infection (ranging from 50.28 to 270.67 μg/ml·h) was significant higher than that of patients without (ranging from 0 to 61.19 μg/ml·h) (96.56 ± 67.52 μg/ml·h vs. 25.81 ± 24.36 μg/ml·h, *t* = 3.14, *p* = 0.005) (Figure 4). The ratio of CSF AUC to serum AUC (AUC_CSF/serum_) in patients with intracranial infection ranged from 15.1 to 80.1% (mean, 33.23 ± 19.31%; median, 26.25%; interquartile range, 20.55–48.35%), and for patients without intracranial infection ranged from 0 to 18.3% (mean, 8.83 ± 8.17%; median, 10.15%; interquartile range, 0.00–17.10%). The AUC_CSF/serum_ of patients with intracranial infection was higher than that of patients without (33.2 ± 19.3% vs. 8.8 ± 8.2%, *t* = 3.72, *p* = 0.001).

The CSF trough concentration was positively correlated with CSF PCT (*r* = 0.503, *p* = 0.017), serum trough concentration (*r* = 0.502, *p* = 0.017), CSF proportion of polynuclear cells (*r* = 0.530, *p* = 0.011), and CSF WBCs/total cells (*r* = 0.608, *p* = 0.003). The CSF trough concentration was negatively correlated with CSF glucose (*r* = −0.464, *p* = 0.030). The CSF trough concentration levels were affected by the serum trough concentration (B: 0.26 ± 0.09, *t* = 2.93, *p* = 0.009), and was mainly affected by the CSF WBCs/total cells (B: 4.81 ± 1.30, *t* = 3.70, *p* = 0.002) (Y = −0.68 + 0.26 × serum trough concentration + 4.81 × CSF [WBCs/total cells]; *R*^2^ = 0.565, *F* = 12.362, *p* < 0.001).

The CSF AUC was positively correlated with CSF WBCs (*r* = 0.437, *p* = 0.042), CSF PCT (*r* = 0.472, *p* = 0.026), serum trough concentration (*r* = 0.426, *p* = 0.048), CSF proportion of polynuclear cells (*r* = 0.471, *p* = 0.027), and CSF WBCs/total cells (*r* = 0.581, *p* = 0.005). The CSF AUC was negatively correlated with CSF glucose (*r* = −0.431, *p* = 0.045). The CSF AUC was not significant correlated with the serum AUC (*r* = 0.323, *p* = 0.143). The CSF AUC levels were affected by the serum trough concentration (B: 5.23 ± 2.36, *t* = 2.22, *p* = 0.039), and were mainly affected by the CSF WBCs/total cells (B: 113.96 ± 35.10, *t* = 3.25, *p* = 0.004) (Y = −17.86 + 5.23 × serum trough concentration + 113.96 × CSF [WBCs/total cells]; *R*^2^ = 0.473, *F* = 8.542, *p* = 0.002).

## Discussion

After intravenous administration, the serum vancomycin concentration changed greatly with obvious peak and trough concentrations. The *T*_*max*_ of the serum concentration was 2 h after the start of vancomycin infusion. And the serum trough concentration appeared half hour before the start of infusion. The *T*_*max*_ of the CSF concentration was later than that of the serum, and appeared 3 h after the start of infusion. Compared with the serum, the concentration–time curve of vancomycin in CSF was gentle and change slightly, with no obvious peak and trough concentrations. Therefore, at any point in time, the CSF extraction and vancomycin concentration measurements can be used to effectively assess. CSF vancomycin concentration was relatively stable, so we confirmed that vancomycin could not easily penetrate the BBB into the central nervous system, and its clearance was also slowly. That might help in maintaining the stability of the CSF drug concentration and effective sterilization effect. Continuous monitoring concentration in CSF at steady-state concentrations is a prerequisite for delineating the concentration curve. To best of our knowledge, the previous report to characterize the gentle fluctuation feature of vancomycin concentration curves in CSF was rare.

The BBB penetration rate is the primary problem for intracranial bacterial infection treatment with intravenous antibiotics. Vancomycin is hydrophilic and macromolecular, which reduce the efficiency of crossing the BBB. Vancomycin enters the central nervous system *via* paracellular pathways rather than active transport mechanisms, and the opening degree of the tight junctions affects its transport speed and volumes (15). The key factor affecting the penetration of vancomycin into CSF is the disruption degree of BBB integrity. The increase in the tight junction gap improves the BBB penetration, making it easier for antimicrobial agents to pass through the BBB.

A clinical trial to show the rate of vancomycin BBB penetration in 22 patients with postsurgical meningitis was carried out by Wang et al. (14) in 2015. They compared the ratio of CSF concentration to serum trough concentration on the day 3 or 4 with 500 mg intravenously every 6 h, and they showed that CSF/serum ratio was 0.291 ± 0.118. Tiede et al. (16) reported the vancomycin CSF/serum ratio was only 7% in nine patients with ventriculitis secondary to subarachnoid hemorrhage. They compared the drug concentration in blood and CSF at 24 and 72 h after the start of continuous intravenously infusion. Vancomycin in CSF has not yet reached the steady-state concentration may be one of the reasons for their low CSF/serum rate. The ratio of the concentration in CSF to the trough concentration in serum was used to evaluate the rate of vancomycin BBB penetration in most previous articles (4, 8, 17, 18), but this parameter may not be accurate for drug exposure. Vancomycin exhibits a time- and concentration-dependent bacterial killing effect. The ratio of CSF AUC to serum AUC was more accurately reflects the permeability of the BBB of vancomycin (19, 20). The multiple measurements of vancomycin required are the main obstacle for calculating AUC in the CSF, and may be the reason for lack of clinical CSF vancomycin AUC study. In the present study, five blood samples and five CSF samples were collected from each case at the fifth intravenous administration, and a simple AUC curve was obtained by calculation. We found that the vancomycin average BBB penetration rate (AUC_CSF/serum_) of patients with intracranial infection was 33.23% and the median was 26.25% in our study. And this two parameters for patients without intracranial infection were only 8.83 and 10.15%. Both the CSF vancomycin AUC and AUC_CSF/serum_ were significantly higher in patients with intracranial infection than those in patients without intracranial infection. Similar to the previous reports, the vancomycin BBB penetration rate was significantly correlated with the degree of intracranial bacterial infection (13, 14), this may be the main reason of individual difference of vancomycin concentration in CSF.

In present study, we found that the CSF trough concentration level could be positively affected by the serum trough concentration, and they had a positive linear correlation (*r* = 0.502, *p* = 0.017). Similarly with our result, several previous studies also reported this correlation. Taheri *et al*., (21) evaluated and compared CSF concentration and serum PK of high-dose vancomycin by continuous infusion *vs*. intermittent infusion in post neurosurgical meningitis patients in 2017. They found that a positive linear correlation was excited between the serum vancomycin trough levels and CSF levels (*r* > 0.970, *p* < 0.001). Cai et al. (8) and Shokouhi et al. (4) also showed this finding with *r* = 0.44 and *r* = 0.60, respectively. In the current study, in addition to serum trough concentration, CSF trough concentration and CSF AUC were positively correlated with CSF PCT, serum trough concentration, CSF proportion of polynuclear cells, and CSF WBCs/total cells. And we also found that CSF trough concentration and CSF AUC were negatively correlated with CSF glucose. To our knowledge, no similar reports have been seen before. These markers are potential indicators for assessing the vancomycin concentration in CSF, and worth further study in the future.

Furthermore, Ishikawa et al. (17) reported that the ratio of CSF protein concentration/serum albumin was a useful marker for estimating the vancomycin CSF/serum ratio (*r* = 0.877, *p* < 0.005), and thought that this could direct the treatment of intracranial infection. Another study carried out by Li et al. (11) showed that the CSF albumin level was a determinant of CSF vancomycin concentration. Unlike note above, we didn't find the significant correlation between CSF protein and CSF vancomycin concentration. The reason may be related to the fact that all the patients underwent surgical operation in our group. CSF protein is one of the important markers for diagnosing intracranial infection and determining the severity of infection, with high sensitivity but low specificity. In addition to infection, blood breaking into the ventricles of the brain is also an important cause of elevated CSF protein levels, which can be maintained for a long time (22).

As noted above, although some studies have reported the quantitative relationships between plasma and CSF vancomycin; and some other studies have focused only on plasma concentrations without systematically assessing exposure. Our results showed that the AUC_CSF_ of vancomycin in patients with postoperative intracranial infection could be predicted by the WBCs to total cells ratio and the serum trough concentration. The increase in the CSF PCT, proportion of polynuclear cells, and WBCs/total cells, and the decrease in the CSF glucose, all indicate aggravation of intracranial infection. Similar to the report of Blassmann et al. (22), we also found no significant correlation between AUC_serum_ and AUC_CSF_. Therefore, compared with the serum AUC, the serum vancomycin trough concentration was more important for the influence on CSF vancomycin concentration. The CSF WBCs/total cells were an important marker of intracranial bacterial infection, and less affected by factors other than infection. Through correlation analysis, we found that the CSF AUC level was affected by the serum trough concentration, and was mainly affected by the CSF WBCs/total cells.

Although it is possible to directly sample the CSF of patients for concentration measurement, the calculation of exposure involves multi-point measurement, and multiple CSF sampling is not only inconvenient to operate, but also add additional financial burden to patients. And not all hospitals in developing countries have therapeutic drug monitoring eligible and technology. A prediction model of vancomycin concentration in CSF established by the serum trough concentration and the CSF WBCs/total cells was presented in the current study, which fully demonstrates the influence of these two factors on vancomycin BBB penetration. It is rare to predict the vancomycin concentration in CSF by combining serum drug trough concentration and CSF infection factors, especially the CSF WBCs/total cells. Our predictive model may be useful for antibiotic selection at the beginning of the treatment and dose adaptation during the treatment when therapeutic drug monitoring is not available.

Six strains were isolated from the CSF of patients with intracranial infection in our small cohort. The vancomycin MIC value of only one strain of *Staphylococcus epidermidis* was 1 μg/ml, and the values of other five strains were all ≤ 0.5 μg/ml. Therefore, we speculated that maintaining the concentration of vancomycin at 4 μg/ml was appropriate for the treatment of intracranial bacterial infection with an MIC ≤ 0.5 μg/ml. Insufficient levels of vancomycin in CSF could delay the disinfection of CSF, and negatively affect the clinical outcome (18). In fact, precise timing of the bacterial clearance of intracranial infection is difficult. Maintaining an adequate concentration of vancomycin in CSF may be more important to shorten the time to sterilize, reduce the occurrence of bacterial drug resistance, and decrease the damage of infection to brain function.

The IDSA updated vancomycin treatment guidelines and no longer recommended the therapeutic trough concentration level, but emphasized the importance of AUC/MIC ≥400 in serum (5). Unfortunately, there is still no recommended concentration of vancomycin in CSF. The CSF AUC/MIC ratios of our 6 patients were isolated with pathogenic bacteria ranged from 72.90 to 196.00 (median, 125.07). Finding the CSF AUC/MIC ratio is what we should do in the future.

Our study has some limitations that should be mentioned. First, the sample size of included cases was small. Second, the sampling frequency of serum and CSF was slightly lower. Third, All patients received a same dose of vancomycin, although we didn't found the remarkable effect of body weight on drug concentration and AUC in the results. Although it may be difficult, further studies are necessary to confirm the present findings, and the fundamental mechanism needs to be studied.

## Conclusions

After intravenous administration of vancomycin, The CSF concentration curve fluctuated gently. The BBB penetration rate of vancomycin in patients with intracranial infection was about 33.23%. The CSF vancomycin concentration in patients with postoperative intracranial infection can be predicted by the WBCs to total cells ratio and the serum trough concentration, and help to adjust the administration of vancomycin.

## Data Availability Statement

The raw data supporting the conclusions of this article will be made available by the authors, without undue reservation.

## Ethics Statement

The studies involving human participants were reviewed and approved by the Human Ethics Committee of the Affiliated Hospital of Qingdao University (QYFY-WZLL-26942). The patients/participants provided their written informed consent to participate in this study.

## Author Contributions

M-CF and WF conceived, designed, and supervised the study. M-CF, J-LS, JS, and NW enrolled patients and collected data. MF and J-WM finalized the analysis. M-CF, WF, and J-LS drafted and revised the manuscript. All authors critically reviewed the manuscript. All authors read and approved the final manuscript.

## Funding

This work was supported, in part, by the National Natural Science Foundation of China (Grant No. 81903872). Author J-LS has received research support from the National Natural Science Foundation of China.

## Conflict of Interest

The authors declare that the research was conducted in the absence of any commercial or financial relationships that could be construed as a potential conflict of interest.

## Publisher's Note

All claims expressed in this article are solely those of the authors and do not necessarily represent those of their affiliated organizations, or those of the publisher, the editors and the reviewers. Any product that may be evaluated in this article, or claim that may be made by its manufacturer, is not guaranteed or endorsed by the publisher.
